# Phenotypic expression of the p.Leu1077Pro *CFTR* mutation in Sicilian cystic fibrosis patients

**DOI:** 10.1186/1756-0500-6-461

**Published:** 2013-11-13

**Authors:** Giuseppe Fabio Parisi, Silvia Cutello, Giovanna Di Dio, Novella Rotolo, Mario La Rosa, Salvatore Leonardi

**Affiliations:** 1 Department of Medical and Pediatric Science, Bronchopneumology and Cystic Fibrosis Unit, University of Catania, Via Santa Sofia 78, Catania 95123, Italy

**Keywords:** Cystic fibrosis, CFTR, pLeu1077Pro, L1077P, Genotype-phenotype, Sicilian patients

## Abstract

**Background:**

The p.Leu1077Pro *CFTR* mutation was firstly described in 1992 as a mild allele that confers a pancreatic sufficiency phenotype but the information collected in database CFTR2 lead to consider p.Leu1077Pro as a severe CF mutation. Although it is typical of Southern Italy, p.Leu1077Pro is not included in the mutation panel firstly tested in individuals originated from this area. The aim of our study was to describe prevalence and clinical features in patients bearing this mutation followed in our Cystic Fibrosis Centre to demonstrate that this mutation should be included in the mutation panel firstly tested in patients originated from Southern Italy.

**Findings:**

We reviewed data from a cohort of 111 cystic fibrosis patients. 4 patients who were heterozygous for the p.Leu1077Pro mutation were included in the study.

In our Cystic Fibrosis Centre, the prevalence of p.Leu1077Pro is 3.6% among all mutations. All patients had positive sweat test values, pancreatic insufficiency and pulmonary exacerbations. One out of four patients even showed both FEV1 and FVC values significantly below the normal range, the presence of bronchiectasis and chronic *Pseudomonas aeruginosa* colonization.

**Conclusions:**

We found that the p.Leu1077Pro CFTR mutation is associated with a classic CF phenotype confirming what is reported in CFTR2 database. The relatively high prevalence of p.Leu1077Pro associated with the severe clinical course of the disease in patients bearing this mutation is of interest for genetic counselling purposes, as it should be part of mutation panel to be tested in individuals originated from Southern Italy.

## Background

Cystic fibrosis (CF) is the most common life-shortening monogenic genetic disease in Caucasians. It is caused by mutations in the cystic fibrosis transmembrane conductance regulator (*CFTR*) gene [NCBI Reference Sequence: NM_000492.3] [1]. There are more than 1900 mutations in *CFTR* and the most prevalent mutation results in deletion of phenylalanine at position 508 (p.Phe508del) [2].

There is a correlation between the general type of *CFTR* mutation and disease phenotype, so the disease severity, to some extent, correlates with organ’s sensitivity to CFTR dysfunction and with the amount of functional protein. Specifically, patients who carry class I-III mutations on both alleles are associated with pancreatic exocrine insufficiency (PI), whereas those carrying at least one class IV-VI *CFTR* mutation are associated with pancreatic sufficiency (PS). These correlations are not absolute and some mutations may be associated with either pancreatic sufficiency or insufficiency [3]. The classic CF phenotype include pathological sweat test, multi-organ involvement with predominant severe respiratory disease, pancreatic insufficiency and male infertility [4,5]. About 10% of patients present a mild form of CF often involving a single organ dysfunction, in many cases with mild respiratory symptoms, pancreatic sufficiency and normal or borderline sweat test [6,7].

The p.Leu1077Pro (L1077P) mutation consists in a transition T to C at nucleotide position 3362 in exon 17b of *CFTR* cDNA that is responsible of change of Leucine (CTG) to Proline (CCG) at position 1077 of the protein. This mutation belongs to the class III *CFTR* mutations and produce a protein that is trafficked to the cell membrane but does not respond to cAMP stimulation [8].

This *CFTR* variant should be predictive of a classic CF phenotype in patients carrying another *CFTR* mutation with no residual function [3] but Bozon et al. [8] firstly found evidence that the p.Leu1077Pro was associated with a pancreatic sufficiency even if their study did not contain functional data.

The information collected in database CFTR2 [9], analyzing data from 40 patients with this mutation, lead to consider p.Leu1077Pro as a severe CF mutation, associated with PI in 78% of patients and *Pseudomonas Aeruginosa* infection in 55% of patients.

Although this mutation is typical of Southern Italy, as evidenced by Castaldo et al. [10] and although it should be predictive of a severe CF phenotype, p.Leu1077Pro is not included in the mutation panel firstly tested in individuals originated from this area since the information collected in CFTR2 are only available from 2011, and the only information held prior to the creation of this database were related to the work of Bozon et al.

The aim of our study was therefore to describe prevalence and clinical features in Sicilian patients bearing the p.Leu1077Pro *CFTR* mutation followed in our Cystic Fibrosis Centre to add new data on this issue.

## Findings

### Patients population

We reviewed data from a cohort of 111 Sicilian patients with a diagnosis of cystic fibrosis followed in the Cystic Fibrosis Centre of the Catania University Hospital (Eastern Sicily - Southern Italy). All patients were followed in the centre since the time of the diagnosis. Sweat test, clinical data and genotypes were available for all the patients. Four patients (3 males, age range 7 – 20 years) who were heterozygous for the p.Leu1077Pro mutation were included in the study: three patients also carried the p.Phe508del (ΔF508) mutation in the second allele while one carried the p.Asn1303Lys (N1303K) mutation. The group included one adult, one adolescent and two children.

Demographic data of the patients are shown in Table 1.

**Table 1:** Demographic data of the patients

**Patient**	**Sex**	**Age**	**BMI**	**Age of diagnosis**	**Genotype**	**Sweat chloride (mmol/L)**	**IRT (ng/ml)**
**1**	M	7 yrs	14.57 (Underweight)	Birth	p.Phe508del/p.Leu1077Pro	112	84/43
**2**	M	10	19.80 (Normal weight)	Birth	p.Phe508del/p.Leu1077Pro	101	155/215
**3**	M	12	19.63 (Normal weight)	Birth	p.Phe508del/p.Leu1077Pro	97	181/100
**4**	F	20	16.44 (Underweight)	4 months	p.Asn1303Lys/p.Leu1077Pro	108	NA

BMI: body mass index.

IRT: immunoreactive trypsinogen, first week/third week after birth. Normal range values <60 ng/ml and <40 ng/ml third week.

Three out of four patients, who were born after 1998, were screened for CF. They had abnormal IRT values and for this reason the CF diagnosis was made soon after birth by sweat chloride values (>60 mmol/l). In the fourth patient the diagnosis was suspected during infant age because of respiratory and gastrointestinal symptoms and then confirmed by the sweat test. The study was approved by our local Committee for Clinical Investigation and informed consent was obtained by each patient or parent.

### Diagnostic tests and clinical data

Sweat chloride concentrations were obtained according to guidelines by the Gibson a Cooke method [11]. Three tests were performed in each patient and the mean value is reported. Blood immunoreactive trypsinogen (IRT) was measured in patients who underwent neonatal screening for CF (in Italy newborn screening is mandatory since 1998) one and three weeks after birth. Pancreatic insufficiency was defined as a faecal elastase <200 mg/g faeces. A quarterly clinical evaluation was performed in our Centre after the diagnosis. During each visit lung function (in patients >6 yrs old) was measured according to the American Thoracic Standard Criteria [12]. Values of forced expiratory volume in 1 s (FEV1) and forced vital capacity (FVC) were expressed as % predicted of normal values adjusted for age, geneder, sex height and weight. The body mass index (BMI) was used for the clinical definition of the nutritional status according to recommendations of international consensus [13,14].

Sputum samples for microbiology were obtained for all adults and adolescents. In younger children who could not expectorate sputum, oropharyngeal swabs were collected. Paranasal sinuses and Chest High Resolution Computed Tomography scan (HRCT) was performed in all patients to assess the presence of rhino/sinus disease or bronchiectasis.

The diagnosis of liver disease was considered if at least one of the following was present in two consecutive evaluations in a period of 6 months: hepatomegaly (liver edge palpable more than 2 cm below the costal margin on the mid clavicular line); elevation of alanine aminotransferase (ALT), aspartate aminotransferase (AST), gamma-glutamyl transpeptidase (GGT) > 1.5 the normal value; ultrasound abnormalities consistent with liver involvement according to conventional criteria. Gallbladder disease was diagnosed if the patient had cholelithiasis or sludging on abdominal sonography [15].

Cystic Fibrosis-related diabetes mellitus (CFRD) was defined by 2-hour glycemia ≥ 200 mg/dl in the OGTT or two fasting glycemia measurements greater than 126 mg/dl [16].

Demographic data included in the study were derived from the last routine evaluation in the Centre. The age at the diagnosis was derived from the date of the first sweat test performed.

### Genetic analysis

Genotyping of *CFTR* was carried out on DNA extracted from blood leucocytes. In our laboratory basic genetic test was performed by reverse dot blot hybridization by using INNO-LiPA CFTR19 and CFTR17 + Tn assays (Innogenetics, Zwiljnaarde, Belgium). The test, performed according to the recommendation of the supplier, allows the detection of the 36 more common mutations in Europe [17]. In patients suspected of CF and a single allele mutation shown by basic genotyping, advanced genetic testing is routinely performed in our laboratory. The p.Leu1077Pro mutation was identified by denaturing high-performance liquid chromatography (d-HPLC) and confirmed by gene sequencing analysis with an automated procedure (Genetic Analyzer, Applied Biosystems). Methods and procedures for dHPLC and DNA sequencing were reported previously [18,19].

## Results

In our Eastern Sicilian Cystic Fibrosis Centre, the prevalence of p.Leu1077Pro is 3.6% among all mutations.

All patients had pancreatic insufficiency treated with enzyme replacement therapy. Patients n.1 and n.4 were underweight with a BMI of 14.57 and 16.44 respectively (Table 1). No hepatobiliary disease or diabetes was recorded (Table 2).

**Table 2:** Clinical data of the patients

**Patient**	**FEV1 (%)**	**FVC (%)**	**SaO**_**2 **_**(%)**	**Bronchiectasis**	**Sputum pathogens**	**Rhino/sinus disease**	**Pancreatic insufficiency**	**Dehydration**
**1**	93.2%	91.7%	97%	No	S. Aureus* P. Aeruginosa**	Yes	Yes	Yes
**2**	89.6%	87.6%	97%	No	S. Aureus* P. Aeruginosa**	No	Yes	No
**3**	96%	95%	99%	No	S. Aureus**	No	Yes	No
**4**	55%	52.5%	96%	Yes	P. Aeruginosa*	Yes	Yes	Yes

*chronically.

**intermittent.

Both adult and children had recurrent respiratory disease with some respiratory exacerbations treated with oral or i.v. antibiotics. Patients n.1 and n.4 had recurrent episodes of rhinosinusitis. Chest HRCT showed the absence of bronchiectasis in all patients except in n. 4 (the older of the group), in which disseminated bilateral bronchiectasis were present.

In Patients n.1, n.2 and n.3 we found FEV1 and FVC values within the normal range of predicted, while patient n.4 showed FEV1 and FVC values below the normal range. Sputum or throat swab cultures revealed chronic presence in Patients n.1 and n.2 and episodic presence in Patient n. 3 of *Staphylococcus aureus*, while the occurrence of *Pseudomonas aeruginosa* in the sputum was reported few times in patients n.1 and n.2 (successfully eradicated by antibiotic treatment) and chronically from 2005 in Patient n.4 (Table 2). Patients n.1 and n.4 had some episodes of dehydration and hypochloronatremia, particularly during the summer, sometimes so severe to require hospitalisation.

## Discussion

The present study provides evidence that the p.Leu1077Pro is a relatively frequent mutation associated with a phenotype characterized by pathological sweat test, pancreatic insufficiency and pulmonary disease.

The p.Leu1077Pro mutation consists in a transition T to C at nucleotide position 3362 in exon 17b of *CFTR* cDNA that is responsible of change of Leucine (CTG) to Proline (CCG) at position 1077 of the protein. This mutation belongs to the III class *CFTR* mutations and produce a protein that is trafficked to the cell membrane but does not respond to cAMP stimulation [8].

Today it is well known that in 90% of patients there is a correlation between the general type of *CFTR* mutation and the phenotype. Specifically, patients who carry class I-III mutations on both alleles are associated with pancreatic exocrine insufficiency, whereas those carrying at least one class IV-VI *CFTR* mutation are pancreatic sufficient [3].

Nevertheless p.Leu1077Pro mutation, although belonging to the class III *CFTR* mutations, was classified by Bozon et al. [8] in one CF patient, carrying p.Phe508del on the other chromosome, as a mild genotype because of its associated pancreatic sufficiency. However, data from CFTR2 [9] seem to contradict what was claimed by Bozon et al. since in 78% of patients analyzed in this database there was pancreatic insufficiency.

Although Cystic Fibrosis is a monogenic disease there is a great clinical diversity among patients, even among those carrying the same mutations in the *CFTR* gene and the degree of organ involvement and the severity of the disease seem to be linked not only to the amount of functional CFTR and to organ’s sensitivity to CFTR dysfunction but also to the influence of both genetic background of patient and environmental factors [20,21].

On this regard, the mutation p.Leu1077Pro is typical of Southern Italy, overall in Puglia, in which the prevalence is higher (1.9% among all mutations) than in the world (< 1%) [10].

In our Eastern Sicilian Cystic Fibrosis Centre its prevalence resulted even higher (3.6% of all mutations) compared to that of Puglia. The influence of ethnic groups of Mediterranean basin and Greek colonisation of Sicily from 750 BC could explain this difference that clinically translates into a greater mixture of genomes within the same territory.

Recently, some modifiers genes, inherited independently by mutations in the *CFTR* gene, have been involved in phenotype/genotype correlation. Some candidate genes are those involved in the immune response and inflammation, because their presence or absence could influence different responses from person to person against bacteria and viruses responsible for respiratory infection, or could modulate the affinity of *Pseudomonas aeruginosa* individual 's respiratory epithelium [22].

Among our patients, three out of four, who were born after 1998, were screened for CF. They had abnormal IRT values and for this reason the CF diagnosis was confirmed soon after birth by sweat test. All of them had a p.Phe508del/p.Leu1077Pro genotype.

In the fourth patient the diagnosis has been performed during infant age, at 4 months of age: the presence of recurrent respiratory and gastrointestinal symptoms were the main reasons to address the child to our Centre to evaluate sweat test which resulted pathological. She had a p.Asn1303Lys/p.Leu1077Pro genotype.

All 4 patients had a pancreatic insufficiency (PI) phenotype and this data, in disagreement with what is reported by Bozon et al. [8], confirm what is evident from CFTR2.

The presence of both pancreatic insufficiency and pathological sweat test seem to indicate that this mutation is responsible for a classical form of CF.

The older patient had several episodes of pneumonia, *Pseudomonas Aeruginosa* chronic lung colonization, disseminated bronchiectasis and a significant reduction of FEV1 and FVC suggesting a severe course of the respiratory disease.

In support of this hypothesis, the other three patients, even if they kept FEV1 and FVC values within normal range, had showed a few pulmonary exacerbations that we treated with oral or i.v. antibiotics. Moreover we found a chronic (in Patients n.1 and n.2) or episodic (in Patients n.3) colonization of *Staphylococcus aureus*, while the occurrence of *Pseudomonas aeruginosa* in the sputum was reported few times in patients n.1 and n.2. In addition, patient n.1 had showed recurrent episodes of rhinosinusitis.

## Conclusions

In conclusion, our study seems to confirm the Consensus Paper [3] and CFTR2 database [9] to the extent that patients who carry class I-III mutations on both alleles are associated with pancreatic exocrine insufficiency. However, given the association with a severe mutation such as p.Phe508del and p.Asn1303Lys, it is clear that only a follow-up could allow definitive conclusions on the course of the pulmonary disease in patients with this genotype/phenotype profile.

The relatively high prevalence of p.Leu1077Pro associated with the severe clinical course of the disease in patients bearing this mutation is of interest for genetic counselling purposes, as it should be part of mutation panel to be tested in individuals originated from Southern Italy.

## Competing interests

All the authors certify that there is no conflict of interest with any financial organization regarding the material discussed in the manuscript.

## Authors’ contributions

GFP and SC revised the literature and wrote the manuscript – GFP, GDD and NR followed the patients in their clinical course. SL conceived the study. MLR and SL made the final analysis and critical revision of the manuscript. All authors read and approved the final manuscript.
